# Nitric Oxide Alleviates Salt Stress Inhibited Photosynthetic Performance by Interacting with Sulfur Assimilation in Mustard

**DOI:** 10.3389/fpls.2016.00521

**Published:** 2016-04-25

**Authors:** Mehar Fatma, Asim Masood, Tasir S. Per, Nafees A. Khan

**Affiliations:** Plant Physiology and Biochemistry Laboratory, Department of Botany, Aligarh Muslim UniversityAligarh, India

**Keywords:** antioxidant, nitric oxide, photosynthesis, salt stress, sulfur

## Abstract

The role of nitric oxide (NO) and sulfur (S) on stomatal responses and photosynthetic performance was studied in mustard (*Brassica juncea* L.) in presence or absence of salt stress. The combined application of 100 μM NO (as sodium nitroprusside) and 200 mg S kg^−1^ soil (S) more prominently influenced stomatal behavior, photosynthetic and growth performance both in the absence and presence of salt stress. The chloroplasts from salt-stressed plants had disorganized chloroplast thylakoids, but combined application of NO and S resulted in well-developed chloroplast thylakoids and properly stacked grana. The leaves from plants receiving NO plus S exhibited lower superoxide ion accumulation under salt stress than the plants receiving NO or S. These plants also exhibited increased activity of ATP-sulfurylase (ATPS), catalase (CAT), ascorbate peroxidase (APX) and glutathione reductase (GR) and optimized NO generation that helped in minimizing oxidative stress. The enhanced S-assimilation of these plants receiving NO plus S resulted in increased production of cysteine (Cys) and reduced glutathione (GSH). These findings indicated that NO influenced photosynthesis under salt stress by regulating oxidative stress and its effects on S-assimilation, an antioxidant system and NO generation. The results suggest that NO improves photosynthetic performance of plants grown under salt stress more effectively when plants received S.

## Introduction

The global aim of increasing agricultural productivity by 70% by the year 2050 for approximately 2.3 billion individuals is facing severe obstructions, primarily due to increasing abiotic stress factors (FAO, Food and Agricultural Organization, 2009). These factors such as cold, drought, flooding, freezing, heat, salinity, or oxidizing agents disturb plant metabolism and negatively impact productivity (Wang et al., 2004; Mian et al., 2011). Salt stress is one of the major abiotic stress factors occupying more than 45 million hectares of irrigated land (Munns and Tester, 2008). It causes excess production of reactive oxygen species (ROS) resulting in induced oxidative stress and inhibition of the Calvin-Benson cycle enzymes (Fatma et al., 2014; Nazar et al., 2014). Plants growing under salt stress develop detoxification mechanisms to avoid damage induced by ROS. These mechanisms are upregulation of activity of enzymatic antioxidants; ascorbate peroxidase (APX), catalase (CAT), glutathione reductase (GR), and superoxide dismutase, and production of non-enzymatic antioxidants; ascorbate (AsA), reduced glutathione (GSH), carotenoids, tocopherol, that help in neutralizing or scavenging ROS (Noctor et al., 2012; Khan and Khan, 2014; Nazar et al., 2015).

Sulfur (S) is the fourth major essential nutrient element with important roles in stress tolerance of plants (Marschner, 1995; Iqbal et al., 2013). Khan et al. (2013) have reported that S is a constituent of major metabolic compounds, such as cysteine (Cys), methionine, GSH, proteins, coenzyme A, sulfo-lipids, iron-sulfur (Fe-S) clusters, glucosinolates, vitamins (biotin and thiamine), and thioredoxin system that have the potential to modulate the physiological processes of plants to alleviate the negative effect of salt stress (Khan and Khan, 2014). Sulfur plays a significant role in the build up of photosynthetic apparatus and electron transport system (Marschner, 1995). The deficiency of S impairs plant metabolism (Honsel et al., 2012) and reduces the chlorophyll content and photosynthesis in *Beta vulgaris* (Kastori et al., 2000). Moreover, its deficiency decreases the photosynthetic efficiency by affecting the content and activity of ribulose 1,5-bisphosphate carboxylase/oxygenase (Rubisco) in *Oryza sativa* (Lunde et al., 2008).

The GSH production is correlated with S-assimilation as both were found up-regulated under conditions of oxidative stress (Noctor et al., 2012). It has been reported that the rate of S-assimilation and GSH biosynthesis were greatly increased in *Brassica napus* plants exposed to saline conditions (Ruiz and Blumwald, 2002), and the increased S supply helped in the protection of *Hordeum vulgare* plants from salt induced-oxidative stress by increasing GSH content (Astolfi and Zuchi, 2013). The exogenous supplementation of GSH in *B. juncea* improved the cell redox state (GSH/GSSG) for better protection and adaptation against salt stress and improvement of photosynthetic capacity (Fatma et al., 2014). Kopriva and Rennenberg (2004) have reported that GSH acted as a signal molecule for S status of plants and was sensitive to ATP-sulfurylase (ATPS), the first enzyme in S-assimilatory pathway (Yi et al., 2010). While Szalai et al. (2009) observed the essential role for GSH in stress tolerance, Mckersie and Leshem (1994) and Gondim et al. (2013) explained that GSH reacts with ^1^O_2_, O_2_^·−^, and ^·^OH ions and functions as a free radical scavenger.

Phytohormones are known to alleviate salt stress by regulating S-assimilation in plants (Fatma et al., 2013). In particular, nitric oxide (NO) has recently been regarded as a potential plant hormone related to plant defense reactions (Gould et al., 2003). The exogenous application of NO improved salt tolerance by alleviating the oxidative damage (Beligni and Lamattina, 2000), stimulating activity of proton-pump and Na^+^/H^+^ antiport in the tonoplast and increasing the K^+^/Na^+^ ratio (Beligni and Lamattina, 2002). It has been reported in *Sorghum bicolor* that NO controls activity of phosphoenolpyruvate carboxylase kinase, and mediates responses of plants to salt stress (Monreal et al., 2013). The improvement of photosynthesis after NO application was due to quenching of excess energy and increase in quantum yield of PSII in *Solanum melongena* seedlings (Wu et al., 2013). Recently, we have shown that NO application enhanced the photosynthetic potential of *B. juncea* under salt stress (Fatma and Khan, 2014). In the presence of stress, NO combines with GSH and forms S-nitrosoglutathione (GSNO) resulting in enhanced S requirement of plants for better survival (Wang et al., 2015a). Studies of Xiong et al. (2010) and Wang et al. (2015b) have shown that NO enhanced tolerance against oxidative stress induced by metals by stimulating GSH biosynthetic pathway.

It is evident from the available literature that the individual application of NO or S improves salt stress tolerance in plants. The alleviation of salt stress with S supplementation involves GSH production, but how NO alleviates salt stress in plants receiving S is not clear. It was, therefore, hypothesized that application of NO in presence of S improved S-assimilation, GSH production, and modulated NO generation to counteract the adverse effects of salt stress on photosynthetic performance of plants. The objectives of the reported research was to study S-assimilation, the antioxidant system and NO generation influenced by NO and S application, and to find out the extent to which photosynthetic performance of salt grown plants was improved.

## Materials and methods

### Plant material, growth conditions and treatments

Healthy seeds of mustard (*B. juncea* L. Czern & Coss. var. Varuna) were surface sterilized with 0.01% HgCl_2_ followed by repeated washings with distilled water and were sown in 23-cm diameter earthen pots containing 5 kg soil with peat and compost (4:1, w/w) mixed with sand (3:1, w/w). The pots were kept under natural day/night conditions with photosynthetically active radiation ~640 μmol m^−2^ s^−1^, average day/night temperature of 22/14 ± 3°C and relative humidity 62–70% in a net house of the Botany Department, Aligarh Muslim University, Aligarh, India. In the experiment, elemental S was used for obtaining 200 mg S kg^−1^ soil (S) by applying 10 days before sowing. Our earlier research has shown that 200 mg S kg^−1^ soil and 100 mg S kg^−1^ soil are excess-S and sufficient-S, respectively, and excess-S promoted photosynthesis and growth more than sufficient-S in the presence of salt through the GSH production (Fatma et al., 2014). NaCl at 100 mM was added to soil before seed sowing for creating salt stress. The addition of 100 mM NaCl develops 10.0 dS m^−1^ salinity (Khan et al., 2009). One hundred ml of NaCl or water was given alternately for 15 days from the sowing time. The concentration of NaCl was selected based on our earlier findings (Fatma et al., 2014). The native soil S concentration was 100 mg S kg^−1^ soil. A concentration of 100 μM NO (as sodium nitroprusside) was applied on foliage of plants alone or on S-grown plants in presence or absence of NaCl with a hand sprayer at 20 days after sowing (DAS). A surfactant teepol (0.5%) was added with the control and NO treatments. Treatments were arranged in a complete randomized block design and number of replicates for each treatment was four (*n* = 4).

### Measurements

#### Chlorophyll content

SPAD chlorophyll meter (SPAD 502 DL PLUS, Spectrum Technologies) was used for the expression of the values of chlorophyll content.

#### Photosynthetic Gas exchange

Net photosynthesis (P_N_), stomatal conductance (g_s_) and intercellular CO_2_ concentration (C_i_) were measured between 11.00 and 12.00 at light saturating intensity on a sunny day in fully expanded uppermost second leaves of plants in each treatment using Infra Red Gas Analyzer (CID-340, Photosynthesis system, Bio-Science, USA). The atmospheric conditions at the time of measurement were: photosynthetically active radiation, ~680 μmol m^−2^ s^−1^; air temperature, ~22°C and relative humidity, ~70%.

#### PS II activity

Chlorophyll fluorometer (Junior-PAM, Heinz Walz, Germany) was used for determining the maximal PS II photochemical efficiency (Fv/Fm) of the fully expanded second leaf from top of the plant. Minimal fluorescence (Fo) and maximal fluorescence (Fm) were obtained by dark adapting the plants for 30 min. The Fo was measured during the weak measuring pulses (125 μmol m^−2^ s^−1^) and a saturating pulse (720 μmol m^−2^s ^−1^) was used to obtain Fm. Variable fluorescence (Fv) was estimated by the difference between Fo and Fm. The quantum yield efficiency of PS II was represented by the ratio of variable fluorescence to maximal fluorescence.

#### Rubisco activity

Rubisco activity was determined by monitoring NADH oxidation at 30°C at 340 nm during the conversion of 3-phosphoglycerate to glycerol 3-phosphate after the addition of enzyme extract to the assay medium (Usuda, 1985). Leaf tissue (1 g) was homogenized in a chilled mortar and pestle with ice-cold extraction buffer that contained 0.25 M Tris-HCl (pH 7.8), 0.05 M MgCl_2_, 0.0025 M EDTA, and 37.5 mg DTT for enzyme extraction. The centrifugation of homogenate was done at 10,000 × g for 10 min at 4°C. The supernatant was used to assay the enzyme. The reaction mixture included 100 mM Tris-HCl (pH 8.0), 40 mM NaHCO_3_, 10 mM MgCl_2_, 0.2 mM NADH, 4 mM ATP, 0.2 mM EDTA, 5 mM DTT, 1 U of glyceraldehyde-3-phosphodehydrogenase and 1 U of 3-phosphoglycerate kinase and 0.2 mM ribulose 1,5-bisphosphate.

#### SDS-PAGE for Rubisco

Protein extraction was done by adopting the method of Carvalho et al. (2005) with slight modifications and Bradford (1976) method was used for the estimation of protein content using bovine serum albumin as standard. Fresh leaf of the plant was taken for the extraction of protein for gel electrophoresis. Leaf tissues (500 mg) were homogenized in extraction buffer (potassium phosphate buffer 0.2 M, pH 8.0) containing: 5 mM EDTA, 5 mM DTT, 0.2 mM phenylmethylsulfonyl fluoride and 50% (w/w) polyvinylpyrrolidone with a small amount of quartz sand. The extracts were centrifuged at 27,000 × g for 10 min at 4°C, and the resulting supernatant was desalted through sephadex PD10 columns Amersham Biosciences equilibrated through the extraction buffer (50 mM, pH 7.5). For all subsequent analyses, the desalted extracts stored at 20°C were used as the source of total soluble protein. Stacking gel of 5% (w/v) and 12.5% in the separation gel (w/v) were used for resolving the protein samples and stained with Coomassie Brilliant Blue R-250 (Sigma) (Laemmili, 1970). Equal amount of 40 μg proteins were loaded onto SDS-PAGE wells uniformly. Molecular weights of protein band were determined in respect to standard protein marker (Merck, pre stained 14.3–97.4 kDa). ImageJ software was used to compare the density/intensity of bands on gel. The density of the bands was presented as relative to the control band.

### Biochemical analyses

#### Determination of Na^+^ and Cl^−^ ions

Root and leaf samples were washed to determine content of Na^+^ and Cl^−^. Plant samples (500 mg) were digested in 19 ml Tri acid mixture, containing 10 ml of 16 M HNO_3_, 5 ml of 18 M H_2_SO_4_ and 4 ml of 11.65 M HClO_4_ in the ratio of 10:5:4 (v/v). The content of ions was extracted in distilled water by boiling for 30 min twice. The filtered extract was used to measure Na^+^ using flame photometer (Khera-391: Khera Instruments, New Delhi), and Cl^−^content was determined by titrating against 0.02 N AgNO_3_ solution using 5% K_2_CrO_4_ as the indicator.

#### Determination of H_2_O_2_ content and lipid peroxidation

The details of the determination of content of H_2_O_2_ have been given in Fatma et al. (2014). Briefly, the content of H_2_O_2_ was determined by adopting the method of Okuda et al. (1991) in leaf tissues (500 mg) grounded in ice-cold 200 mM HClO_4_. This was centrifuged at 1200 × g for10 min followed by neutralization of HClO_4_ of the supernatant with 4 M KOH. The insoluble KClO_4_ was eliminated by further centrifugation at 500 × g for 3 min. In a final volume of 1.5 ml, the reaction mixture contained 1 ml of the eluate, 400 μl of 12.5 mM 3-(dimethylamino) benzoic acid in 0.375 M phosphate buffer (pH 6.5), 80 μl of 3-methyl-2-benzothiazoline hydrazone and 20 μl of peroxidase (0.25 Unit). The reaction was started by the addition of peroxidase at 25°C and the increase in absorbance was recorded at 590 nm.

Lipid peroxidation was expressed as the content of thiobarbituric acid reactive substances (TBARS) and was estimated using the method of Dhindsa et al. (1981). Leaf tissues (500 mg) were ground in 0.25% 2-thiobarbituric acid in 10% trichloroacetic acid and the mixture was heated at 95°C for 30 min and cooled quickly on ice bath. This was followed by centrifugation at 10,000 × g for 10 min. Four ml of 20% trichloroacetic acid containing 5% thiobarbituric acid was added to 1 ml aliquot of the supernatant. The intensity of the color was read at 532 nm.

#### Accumulation of superoxide ion

The accumulation of superoxide ion (${\text{O}}_{2}^{-}$) in leaves was noted by histochemical staining method by adopting the method of Wang et al. (2011) with slight modification using nitro blue tetrazolium (NBT). The samples (3 leaves) from each treatment were immersed into 1 mg/ml NBT solution prepared in 10 mM phosphate buffer (pH 7.8) at ambient temperature (23°C) under day light for 6 h. The blue spots on NBT staining appeared, which were cleared in concentrated ethanol and then kept in 70% ethanol. The pictures were taken with a NIKON digital camera (COOLPIX110).

### Antioxidant metabolism

#### Assay of antioxidant enzymes

The method of Aebi (1984), Nakano and Asada (1981), and Foyer and Halliwell (1976) with slight modifications, were adopted for the measurement of CAT, APX, and GR activity, respectively. Fresh leaf tissues (200 mg) were homogenized in chilled mortar and pestle with an extraction buffer containing 0.05% (v/v) Triton X-100 and 1% (w/v) polyvinylpyrrolidone in 100 mM potassium-phosphate buffer (pH 7.0). At 4°C, the homogenate was centrifuged at 15,000 × g for 20 min. The supernatant obtained after centrifugation was used for the assay of CAT (EC; 1.11.1.6) and GR (EC; 1.6.4.2). For the assay of APX (EC; 1.11.1.11) extraction buffer was supplemented with 2 mM AsA. The details of procedure have been described earlier in our studies (Fatma et al., 2014).

### S-assimilation

#### Activity of ATPS and Cys content

The method of Lappartient and Touraine (1996) was adopted for the measurement of ATPS activity. The activity of ATPS was assayed *in vitro* in leaves by measuring molybdate-dependent formation of pyrophosphate.

Content of Cys in leaves was determined spectrophotometrically adopting the method of Gaitonde (1967). The amount of Cys was calculated with reference to a calibration curve obtained under similar conditions for standard Cys covering a range of 5–20 nmol. The details of the determination have been described earlier (Fatma et al., 2014).

#### S content

Oven-dried leaf powder (100 mg) was taken in 75 ml digestion tube. In the tube, 4 ml acid mixture (contained concentrated HNO_3_ and HClO_4_ in the ratio of 85:1, v/v) and 7.5 mg of selenium dioxide as catalyst were added. The mixture was digested and the volume of the colorless solution was made up to 75 ml with deionized water. The interference of silica was checked by filtering the contents of the tube. A 5 ml aliquot was pipette out from the digested solution for turbidity development in 25 ml volumetric flask. Turbidity was developed by adding 2.5 ml gum acacia (0.25%) solution, 1.0 g BaCl_2_ sieved through 40–60 mm mesh and the volume was made up to the mark with deionized water. The contents of 25 ml volumetric flask were thoroughly shaken till BaCl_2_ completely dissolved. Turbidity was allowed to develop for 2 min. The values were recorded at 415 nm within 10 min after the turbidity development. A blank was also run simultaneously after each set of determination. The amount of sulfate was calculated with the help of a calibration curve drawn a fresh using a series of K_2_SO_4_ solutions. The method has been given earlier (Fatma et al., 2014).

#### GSH content and redox state

The determination of GSH was done using the method of Griffith (1980). Reduced glutathione was assayed by an enzyme recycling procedure in which it was sequentially oxidized by 5, 5′-dithiobis-2-nitrobenzoic acid and reduced by NADPH in the presence of GR. GSH was masked by derivatization with 2-vinylpyridine for the assay of GSSG. Fresh leaf tissues (500 mg) were ground in liquid nitrogen using mortar and pestle and suspended in 2 ml of 5% (w:v) sulfosalicylic acid and then centrifuged at 12,000 × g for 10 min. The details for the determination of GSH and redox state have been elaborated in Fatma et al. (2014).

#### NO generation

Generation of NO was determined by estimating nitrite content adopting the method previously described by Zhou et al. (2005) with minor modifications. Homogenization of leaves (500 mg) was done in 3 ml of 50 mM ice cold acetic acid buffer (pH 3.6) containing 4% zinc acetate using mortar and pestle. Thereafter, the homogenate was centrifuged at 11,500 × g for 15 min at 4°C and the supernatant was collected. The pellet was washed with 1 ml of the extraction buffer and again centrifuged. Supernatants from the two spin were combined and neutralized by adding 100 mg of charcoal. The filtrate was leached and collected, after vortex and filtration. One ml each of the filtrate and Greiss reagent (1% sulphanilamide and 0.1% N-1-napthylethylenediaminedihydrochloride in 5% H_2_PO_4_ solution) mixed in the ratio (1:1) was incubated at room temperature for 30 min. Finally, the absorbance of the reaction mixture was taken at 540 nm and NO content was estimated from a calibration curve plotted using sodium nitrite as standard.

#### Abscisic acid determination

The content of abscisic acid (ABA) was determined according to the method previously described by Hung and Kao (2003) with slight modifications. Leaves were frozen with liquid nitrogen immediately and ground into fine powder. Then, the powder was homogenized in the extraction solution (80% methanol containing 2% glacial acetic acid) using a motor and pestle. The crude extract was centrifuged and passed through polyvinylpyrrolidone column and C_18_ cartridges to remove plant pigments and other non-polar compounds which could interfere in the immunoassay. The eluates were then concentrated to dryness by vacuum evaporation and resuspended in Tris-buffered saline before enzyme-linked immunosorbent assay (ELISA). Subsequently, ABA was determined with ABA immunoassay detection kit (PGR-1; Sigma-Aldrich, St. Louis, MO, USA) as per the user manual. The values were recorded at 405 nm and the ABA content was estimated from a calibration curve plotted by using standard ABA.

### Structural and chloroplast ultrastructure studies

#### Confocal microscopy

For confocal microscopy young axillary leaves of plant was plucked and dried in a desiccator. Thereafter, the leaves were processed from the dorsal side to remove the epidermal layer and expose the stoma. The leaves were fine sectioned and mounted on glycerol cover-slip on glass slides. The samples were then analyzed under the Olympus Fluoview TM-FV1000 confocal microscope at 60X magnification and 1X optical zoom. DIC images were captured for different samples keeping fixed microscopy parameters. Fluoview FV10 ver 1.7 was used to analyze samples and scale bars were used.

#### Scanning electron microscopy

Leaf samples were prepared for scanning electron microscopy (SEM) by adopting the method of Daud et al. (2009) with slight modifications. Fresh leaves samples were taken from the axillary positions (ideally the leaf was 1.5 × 1.5 inch in size) and were preferably air dried in dessicator. Subsequently, leaf samples were first fixed with 2.5% glutaraldehyde plus 2% paraformaldehyde in 0.1 M phosphate buffer (pH 7.0) in equal quantity for more than 4 h and then washed three times with phosphate buffer for 15 min at each step. The samples were then post fixed with 1% osmium oxide in phosphate buffer (pH 7.0) for 1 h and washed three times with the same phosphate buffer for 15 min. After that, firstly, the specimens were dehydrated by a graded series of ethanol (50, 70, 80,90, 95, and 100%) for about 15–20 min at each step, transferred to the mixture of alcohol and iso-amyl acetate (v/v = 1) for about 30 min. Then, the samples were transferred to pure iso-amyl acetate for 1 h. In the end, the specimens were dehydrated in Carl Zeiss EVO 40 (Germany) scanning electron microscope critical point dryer with liquid CO_2_. The dehydrated specimen was coated with gold-palladium and observed under the Carl Zeiss EVO 40 (Germany) scanning electron microscope at extra high tension or high voltage at 20 kV and magnification of 1500 X or 5000 X. The stomata were observed under the scanning electron microscope at 1.50 K X and 5.0 K X. The stomatal frequency was determined by counting the number of stomata in the microscope field of view.

#### Transmission electron microscopy

Leaf tissues for chloroplast ultrastructure were prepared for transmission electron microscopy (TEM) by adopting the method of Sandalio et al. (2001) with slight modifications. Leaf samples were cut with razor blade into 1 mm^2^ segments and fixed in 2.5% glutaraldehyde solution in 50 mM phosphate buffer (pH 6.8) for 2.5 h at room temperature. Leaf tissue was then post-fixed for 30 min in 1% osmium tetroxide in 50 mM sodium cacodylate buffer (pH 7.2) and dehydrated in ethanol graded series (30–100%, v/v). After dehydration in a graded series of ethanol, replaced to propylene oxide, and then the tissue was embedded in Spurr resin. Ultrathin sections were taken by using Leica EM UC6 ultramicrotome. Sections were stained with uranyl acetate and lead citrate and examined using a transmission electron microscope (JEOL 2100F, JAPAN) accelerating voltage at 120 kV and magnification at 6000 X and 1200 X. The chloroplast ultrastructure (thylakoid membranes) was observed from TEM images.

#### Growth characteristics

From each pot, plants were uprooted carefully and washed to remove dust. Leaf area was measured with the help of leaf area meter (LA 211, Systronics, New Delhi, India).

After drying the sample in a hot air oven at 80°C till constant weight, the plants were recorded for obtaining dry mass.

#### Statistical analysis

Data were analyzed statistically using analysis of variance (ANOVA) by SPSS 17.0 for Windows, and presented as treatment mean ± SE (*n* = 4). Least significant difference (LSD) was calculated for the significant data at *P* < 0.05. Bars showing the same letter are not significantly different by LSD test at *P* < 0.05.

## Results

### Effect of NO and S on photosynthetic performance

The plants receiving NO or S showed higher values for P_N_, g_s_, C_i_, PSII activity and Rubisco activity compared to control plants in the absence or presence of salt. Application of NO or S in plants without salt equally increased P_N_ by 66.0%, g_s_ by 31.0%, C_i_ by 38.0%, maximal PS II photochemical efficiency by 18.0% and Rubisco activity by 38.0% in comparison to control plants. In presence of salt, NO or S increased P_N_, g_s_, C_i_, maximal PS II photochemical efficiency and Rubisco activity by 12.1% or 35.6%, 9.6% or 21.6%, 12.5% or 25.9%, 5.5% or 12.3%, and 11.1% or 20.8%, respectively compared to control plants (Table 1). Application of NO and S together resulted in maximum values of the above observed photosynthetic parameters in plants grown with or without salt compared to control. In the presence of salt, plants receiving NO and S together alleviated the effects of salt stress and enhanced P_N_, g_s_, Ci, maximal PS II photochemical efficiency and Rubisco activity by 80.3, 47.1, 52.2, 24.7, and 72.2%, respectively compared to control plants (Table 1).

**Table 1:** **Chlorophyll content, maximal PS II photochemical efficiency, Rubisco activity, stomatal conductance, intercellular CO_**2**_ concentration, and net photosynthesis of mustard (***Brassica juncea*** L.) leaves treated with 100 μM nitric oxide (NO) and/or grown with S (200 mg S kg^**−1**^ soil; S) in presence or absence of 100 mM NaCl at 30 DAS**.

**Treatments**	**Chlorophyll content (SPAD value)**	**Maximal PS II photochemical efficiency**	**Rubisco activity (μmol CO_2_ mg^−1^ protein min^−1^)**	**Stomatal conductance (mmol CO_2_ m^−2^ s^−1^)**	**Intercellular CO_2_ concentration (μmol CO_2_ mol^−1^)**	**Net photosynthesis (μmol CO_2_ m^−2^ s^−1^)**
0	32.7 ± 1.18^f^	0.73 ± 0.015^f^	0.72 ± 0.030^f^	365 ± 11.1^f^	255 ± 10.4^f^	13.2 ± 0.61^f^
NaCl	22.7 ± 1.11^g^	0.58 ± 0.014^g^	0.43 ± 0.028^g^	280 ± 09.3^g^	173 ± 07.3^g^	7.8 ± 0.55^g^
S	42.1 ± 1.62^c^	0.87 ± 0.018^c^	1.02 ± 0.051^c^	483 ± 18.7^c^	360 ± 14.7^c^	22.2 ± 0.75^c^
NO	41.2 ± 1.60^c^	0.86 ± 0.018^c^	0.99 ± 0.047^c^	479 ± 18.5^c^	352 ± 14.1^c^	21.9 ± 0.71^c^
S + NO	50.3 ± 1.85^a^	0.97 ± 0.019^a^	1.37 ± 0.039^a^	578 ± 22.3^a^	428 ± 15.5^a^	26.5 ± 0.90^a^
S + NaCl	38.0 ± 1.55^d^	0.82 ± 0.018^d^	0.87 ± 0.040^d^	444 ± 17.0^d^	321 ± 13.7^d^	17.9 ± 0.68^d^
NO + NaCl	35.0 ± 1.45^e^	0.77 ± 0.017^e^	0.80 ± 0.030^e^	400 ± 14.0^e^	287 ± 11.9^e^	14.8 ± 0.67^e^
S+ NO+ NaCl	46.2 ± 1.70^b^	0.91 ± 0.019^b^	1.24 ± 0.035^b^	537 ± 20.8^b^	388 ± 15.1^b^	23.8 ± 0.81^b^

*Data are presented as treatments mean ± SE (n = 4). Data followed by same letter are not significantly different by LSD test at P < 0.05. Rubisco, ribulose-1,5-bisphosphate carboxylase/oxygenase*.

### SDS-PAGE for Rubisco

**Figure 1:**
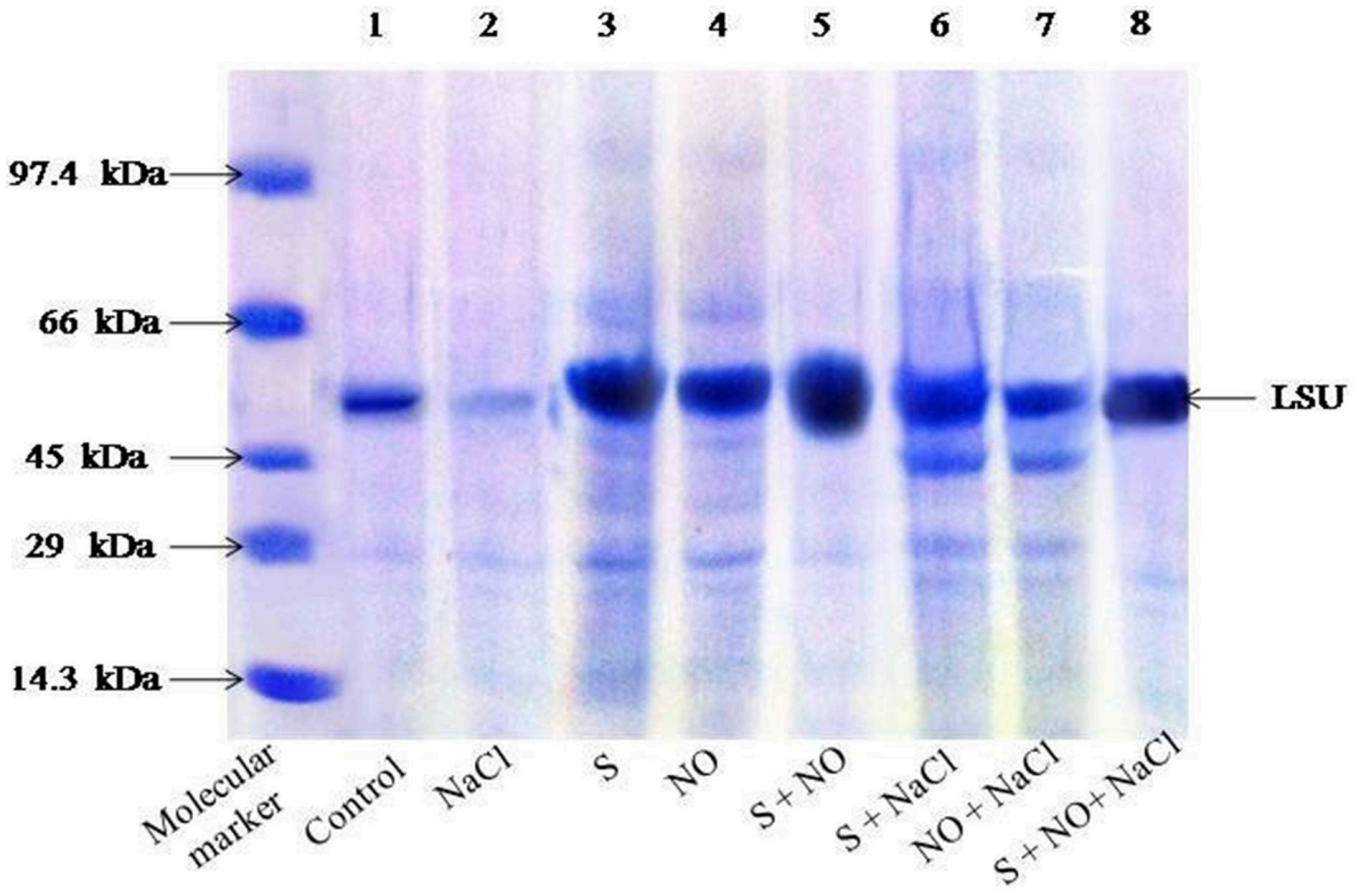
**SDS-PAGE protein profile of Rubisco of mustard ***(Brassica juncea*** L.) leaves treated with 100 μM nitric oxide (NO) and grown with S (200 mg S kg^**−1**^ soil; S) in presence or absence of 100 mM NaCl at 30 DAS**. Equal amount of protein (40 μl) were loaded on to each lane. The proteins expressed in the range of 14.3–97.4 kDa. LSU, large subunits of Rubisco (native soil S content: 100 mg S kg^−1^ soil).

SDS-PAGE showed differences in the intensity of Rubisco protein band of plants grown with salt or plants treated with NO and S in absence or presence of salt (Figure 1). Salt treatment resulted in degradation of protein and thus low intensity of protein band. On the other hand, plants receiving NO or S exhibited distinct protein band, but maximal increase in the density/intensity of protein band was obtained with the combined treatment of NO plus S. The effect of S was found more pronounced than NO in increasing the protein band intensity (Figure 1). The relative density of all bands was calculated with respect to control. The density of bands decreased by 45% under salt stress, while the density of bands increased by 31 and 23%, respectively with S or NO in comparison to control. The combined treatment of NO and S increased the density of bands maximally by 43% in comparison to control in absence of salt. In presence of salt, S and NO increased the band density by 34% in comparison to control.

### Effect of NO and S on oxidative stress

The content of Na^+^ and Cl^−^ increased in roots and leaves in salt treated plants (Table 2). Application of NO or S resulted in reduction of Na^+^ and Cl^−^ content in both roots and leaves under no stress compared to control plants. Under salt stress, S application proved better than NO in lowering content of Na^+^ and Cl^−^ in comparison to control plants. However, the reductions in the content of ions were greatest when plants were supplemented with NO plus S under both salt stress and no stress conditions.

**Table 2:** **Root and leaf Na^**+**^ and Cl^**−**^ content and H_**2**_O_**2**_ and TBARS content in mustard (***Brassica juncea*** L.) leaves treated with 100 μM nitric oxide (NO) and/or grown with S (200 mg S kg^**−1**^ soil; S) in presence or absence of 100 mM NaCl at 30 DAS**.

**Treatments**	**Root Na^+^**	**Root Cl^−^**	**Leaf Na^+^**	**Leaf Cl^−^**	**H_2_O_2_**	**TBARS**
	**(mg g**^**−1**^ **leaf dry mass)**				**(nmol g**^**−1**^ **leaf dry mass)**	
0	8.80 ± 0.808^e^	7.5 ± 0.340^e^	8.9 ± 0.612^e^	7.2 ± 0.303^e^	18.7 ± 0.989^b^	5.00 ± 0.220^b^
NaCl	27.30 ± 0.412^a^	18.1 ± 0.430^a^	22.5 ± 1.000^a^	14.9 ± 0.786^a^	33.6 ± 0.988^a^	10.10 ± 0.234^a^
S	7.60 ± 1.077^f^	5.5 ± 0.306^f^	6.9 ± 0.532^f^	5.2 ± 0.410^f^	10.4 ± 1.020^e^	3.10 ± 0.180^e^
NO	7.80 ± 1.064^f^	5.7 ± 0.309^f^	7.3 ± 0.661^f^	5.5 ± 0.444+	11.2 ± 1.090^e^	3.30 ± 0.190^e^
S + NO	6.20 ± 0.602^g^	4.2 ± 0.288^g^	5.4 ± 0.560^g^	4.2 ± 0.209^g^	6.1 ± 0.870^g^	1.93 ± 0.140^g^
S + NaCl	13.70 ± 0.434^c^	11.3 ± 0.555^c^	13.8 ± 0.888^c^	9.8 ± 0.530^c^	14.2 ± 1.010^d^	3.80 ± 0.190^d^
NO + NaCl	15.20 ± 0.596^b^	12.2 ± 0.612^b^	16.5 ± 0.987^b^	12.5 ± 0.612^b^	16.2 ± 1.090^c^	4.40 ± 0.208^c^
S + NO+ NaCl	11.10 ± 0.707^d^	8.8 ± 0.303^d^	10.9 ± 0.732^d^	8.5 ± 0.660^d^	8.0 ± 0.920^f^	2.80 ± 0.170^f^

*Data are presented as treatments mean ± SE (n = 4). Data followed by same letter are not significantly different by LSD test at P < 0.05. Cl^−^, chloride ion; H_2_O_2_, hydrogen peroxide; Na^+^, sodium ion; TBARS, thiobarbituric acid reactive substances*.

Salt stress increased content of H_2_O_2_ and TBARS equally by about two-times compared to control plants. The individual application of S and NO reduced the content of H_2_O_2_ and TBARS by about 40 and 30% under no stress, but these reductions were about 24 and 12%, respectively under salt stress compared to control plants. The maximal reduction in content of H_2_O_2_ and TBARS was found with the combined treatment of NO and S under no stress and salt stress in comparison to control plants (Table 2).

**Figure 2:**
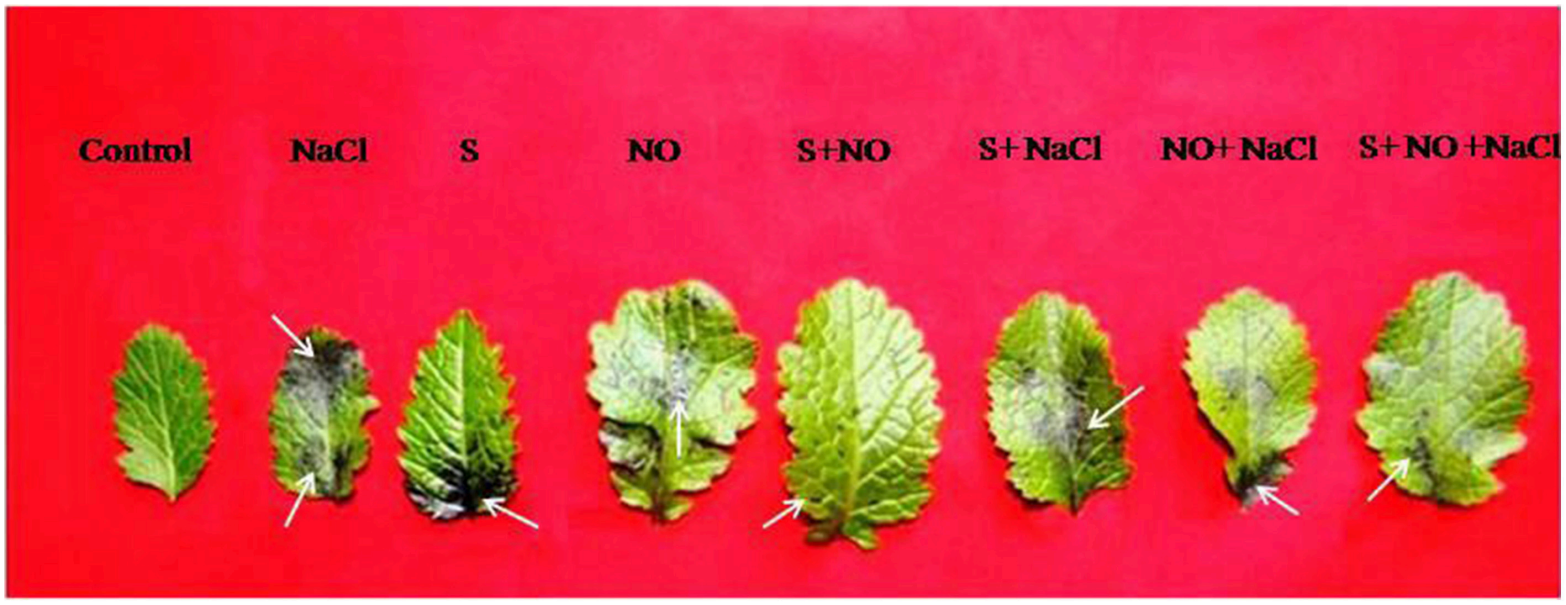
*****In situ*** accumulation of superoxide ion (${\text{O}}_{2}^{-}$) by nitro blue tetrazolium (NBT) staining of mustard (***Brassica juncea*** L.) leaves after dehydration**. The leaves originated from plants treated with/without 100 mM NaCl, S (200 mg S kg^−1^ soil; S) or 100 μM nitric oxide (NO) individually or in combinations at 30 DAS. Arrow (→) shows ${\text{O}}_{2}^{\cdot -}$ accumulation.

Histochemical staining with NBT to demonstrate the ${\text{O}}_{2}^{-}$ accumulation showed that leaves were scarcely stained by NBT in the absence of dehydration (Figure 2), but were blue stained (marker for ${\text{O}}_{2}^{-}$ accumulation) when the leaves were exposed to dehydration for 6 h. The leaves from the salt treated plants showed deepest staining, while NO plus S treatment resulted in least staining compared to control plants. NO or S treatment resulted in shallow staining compared to control plants. Under salt stress, plants treated with NO plus S showed lesser staining than their individual treatments and salt treated plants. The effect of S was more prominent in reducing the accumulation of ${\text{O}}_{2}^{-}$ than NO in presence of salt.

### Effect of NO and S on antioxidant enzymes

**Figure 3:**
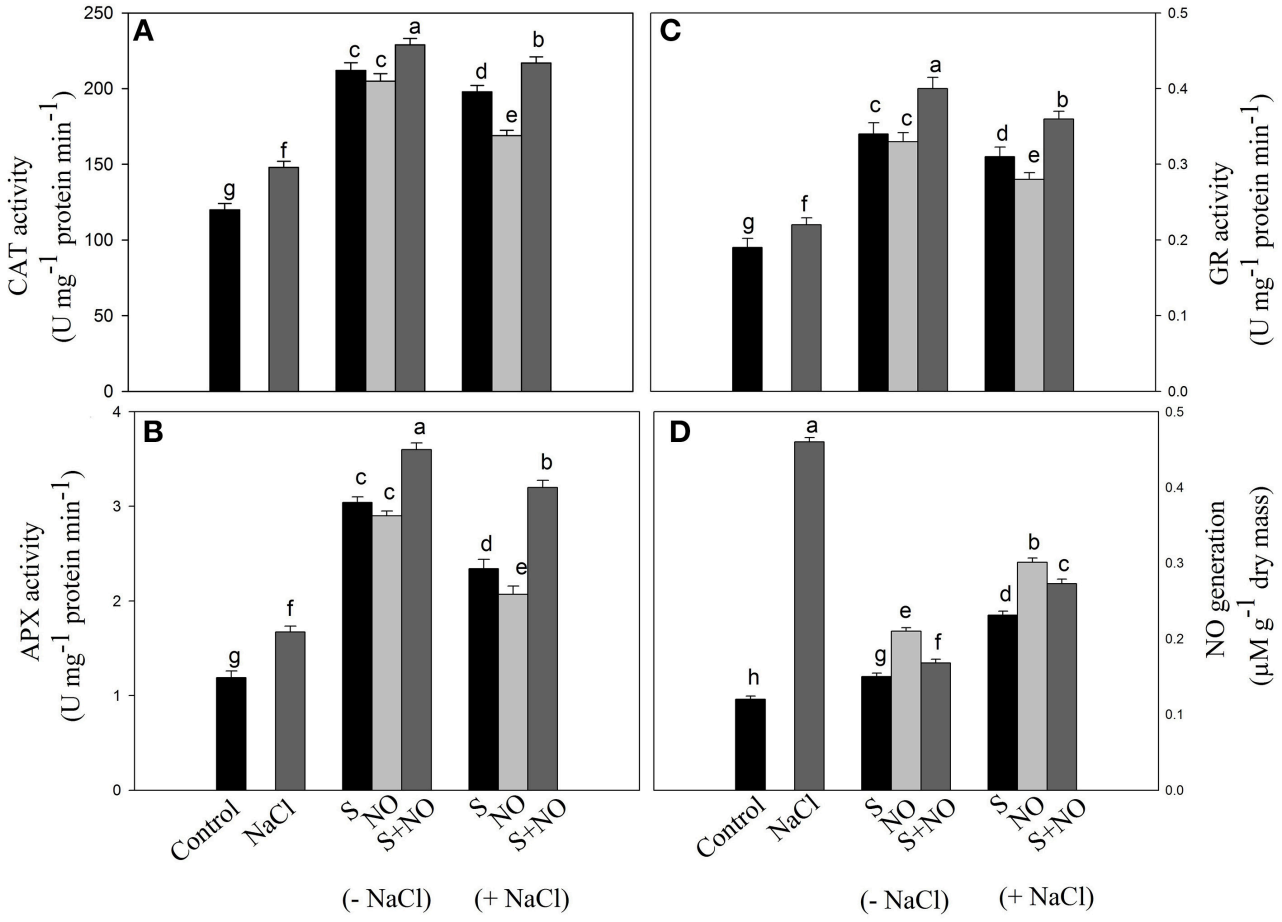
**Activity of CAT (A), APX (B), GR (C), and NO generation (D) in mustard (***Brassica juncea*** L.) leaves treated with 100 μM nitric oxide (NO) and/or grown with S (200 mg S kg^**−1**^ soil; S) in presence or absence of 100 mM NaCl at 30 DAS**. Data are presented as treatments mean ± SE (*n* = 4). Data followed by same letter are not significantly different by LSD test at *P* < 0.05. APX, ascorbate peroxidase; CAT, catalase; GR, glutathione reductase.

The activity of CAT, APX, and GR was assayed in order to evaluate the extent to which the antioxidant enzymes system was influenced by salt stress and how they were modulated by NO and S. Salt stress increased the activity of these enzymes compared to control plants. NO or S increased the activity of CAT and GR equally by about 73%, and APX activity by about 2.5-times under no stress compared to control plants. In salt grown plants, NO and S individually increased the activity of CAT by 65 and 40.8%, APX by 96.6 and 73.9% and GR by 63.1 and 47.3%, respectively in comparison to control plants. However, maximum increase in the activity of these enzymes was noted with the combined treatment of NO and S under both no stress and salt stress conditions (Figures 3A–C).

### Effect of NO and S on S-assimilation and redox state (GSH/GGSG)

The influence of NO or S was assessed on S-assimilation to monitor its contribution in salt stress alleviation. Salt stress decreased S content (−14.2%) and redox state (−56.8%), but increased the activity of ATPS (+13.3%) and accumulation of Cys (+15.2%) and GSH (+21%) in comparison to control. The decrease in S content and redox state by salt stress was recovered by the application of NO or S almost equally by 50% compared to control plants. Moreover, application of NO or S also equally increased the activity of ATPS by 83%, Cys content by 55% and GSH content by 70% compared to control plants under no stress. In salt stressed plant, GSH content was higher than the control plants suggesting that plants used the available S for GSH synthesis resulting in lower values of S content. Plants treated with S under salt stress exhibited increased ATPS activity (+61.6%), Cys content (+45.7%), S (+24.4%), GSH content (+49.1%), and redox state (+25.7%) in comparison to control plants. However, a lesser increase in ATPS activity (+47.5%), Cys content (+28.8%), S content (+12.2%), and GSH content (+36.8%) and redox state (+11.6%) was noted with NO in salt treated plants. Nevertheless, supplementation of NO plus S to plants grown with salt or no stress condition resulted in maximum increase in ATPS activity (+158.3 and +200%), Cys content (+72.8 and +84.7%), S (+71.4 and +79.5%), GSH (+87.7 and +96.4%) and redox state (+88.3 and +97.9%), respectively compared to control plants (Table 3).

**Table 3:** **Activity of ATPS, content of Cys, S and GSH, and redox state (GSH/GSSG) in mustard (***Brassica juncea*** L.) leaves treated with 100 μM nitric oxide (NO) and/or grown with S (200 mg S kg^**−1**^ soil; S) in presence or absence of 100 mM NaCl at 30 DAS**.

**Treatments**	**ATPS (μmol g^−1^ protein s^−1^)**	**Cys (nmol g^−1^ leaf dry mass)**	**S (mg g^−1^ leaf dry mass)**	**GSH (nmol g^−1^ leaf dry mass)**	**Redox state (GSH/GSSG)**
0	1.20 ± 0.064^g^	5.9 ± 0.290^g^	4.9 ± 0.232^f^	57 ± 2.20^g^	19.80 ± 0.612^f^
NaCl	1.36 ± 0.043^f^	6.8 ± 0.298^f^	4.2 ± 0.119^g^	69 ± 2.09^f^	8.55 ± 0.643^g^
S	2.27 ± 0.707^c^	9.2 ± 0.312^c^	7.5 ± 0.311^c^	99 ± 2.57^c^	32.00 ± 0.745^c^
NO	2.20 ± 0.820^c^	9.1 ± 0.308^c^	7.3 ± 0.330^c^	95 ± 2.45^c^	31.30 ± 0.732^c^
S+NO	3.60 ± 0.088^a^	10.9 ± 0.442^a^	8.8 ± 0.230^a^	112 ± 2.80^a^	39.20 ± 0.989^a^
S + NaCl	1.94 ± 0.059^d^	8.6 ± 0.307^d^	6.1 ± 0.313^d^	85 ± 2.33^d^	24.90 ± 0.666^d^
NO + NaCl	1.77 ± 0.065^e^	7.6 ± 0.301^e^	5.5 ± 0.300^e^	78 ± 2.10^e^	22.10 ± 0.654^e^
S + NO + NaCl	3.10 ± 0.081^b^	10.2 ± 0.370^b^	8.4 ± 0.278^b^	107 ± 2.73^b^	37.30 ± 0.987^b^

*Data are presented as treatments mean ± SE (n = 4). Data followed by same letter are not significantly different by LSD test at P < 0.05. ATPS, ATP-sulfurylase; Cys, cysteine; GSH, reduced glutathione; GSSG, oxidized glutathione*.

### Effect of NO and S on NO generation

Plants grown with salt showed increased NO generation by 3.8-times compared to control plants, but exogenously applied NO resulted in reduced NO generation by 2.2-times compared to salt treated plants. However, maximum reduction in NO generation by 3-times was noted with S application compared to salt treated plants under no stress. The combined application of NO plus S decreased NO formation by 2.2-times compared to salt treated plants. Under salt stress, plants receiving NO or S reduced NO generation by 1.5 or 2-times, respectively compared to salt treated plants. The combination of NO and S in the presence of salt reduced NO generation by 1.7-times compared to salt-treated plants (Figure 3D).

### Effect of NO and S on ABA content

**Figure 4:**
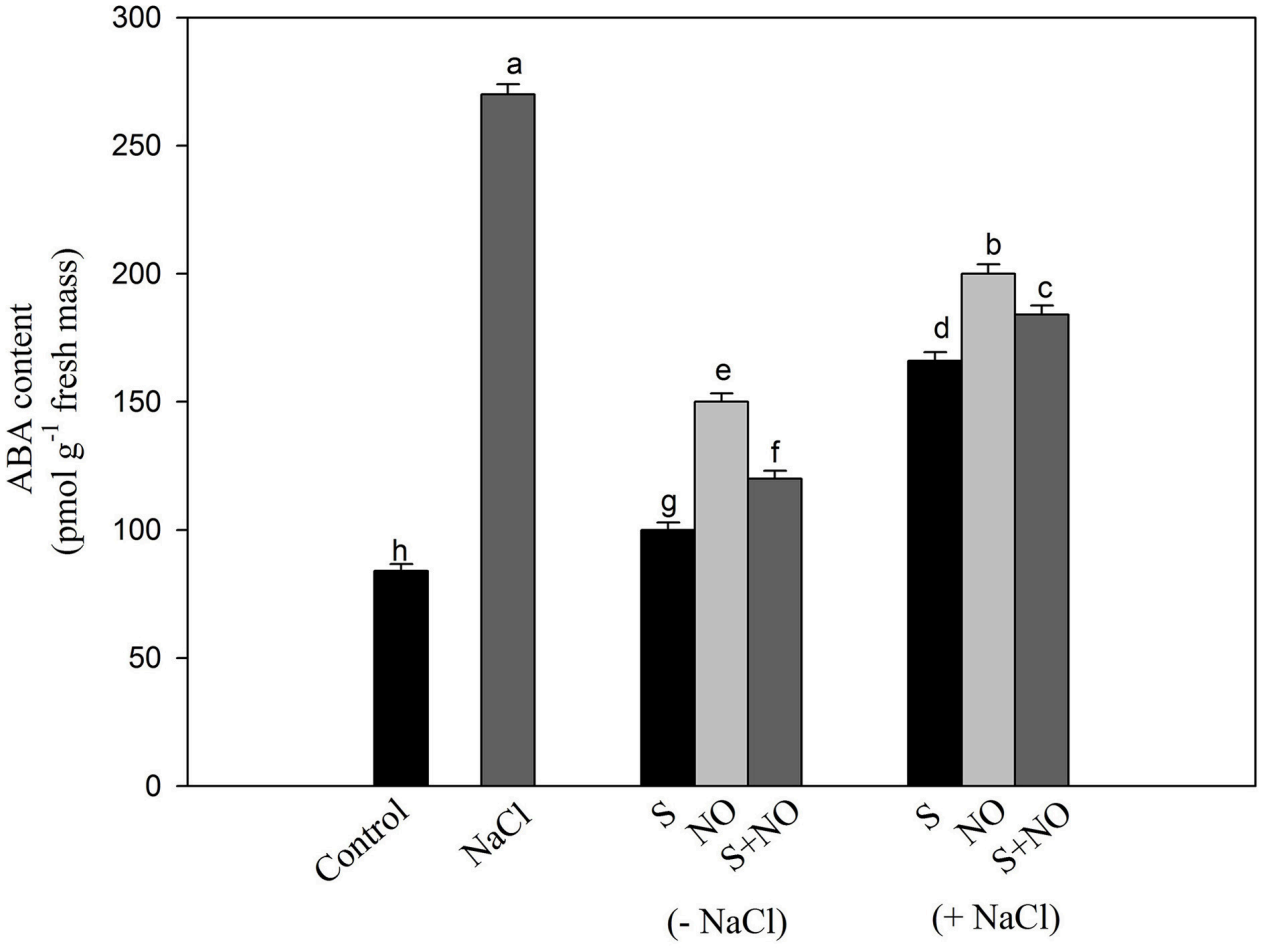
**Content of ABA in mustard (***Brassica juncea*** L.) leaves treated with 100 μM nitric oxide (NO) and/or grown with S (200 mg S kg^**−1**^ soil; S) in presence or absence of 100 mM NaCl at 30 DAS**. Data are presented as treatments mean ± SE (*n* = 4). Data followed by same letter are not significantly different by LSD test at *P* < 0.05.

The content of ABA was influenced by NO and S given alone or in combination. Supplementation of plants with NO resulted in 1.7-times greater ABA accumulation than control plants. Plants given S treatment showed 2.7-times lesser ABA content compared to control plants. The combined application of NO and S also decreased ABA content compared to salt treated plants under no stress. Plants receiving NO or S under salt stress exhibited reduced ABA content by 1.3 or 1.6-times, respectively compared to salt treated plants. In salt treated plants, NO plus S decreased the ABA content by 1.4-times compared to salt treated plants (Figure 4).

### Effect of NO and S on stomatal response

#### Stomatal response

**Figure 5:**
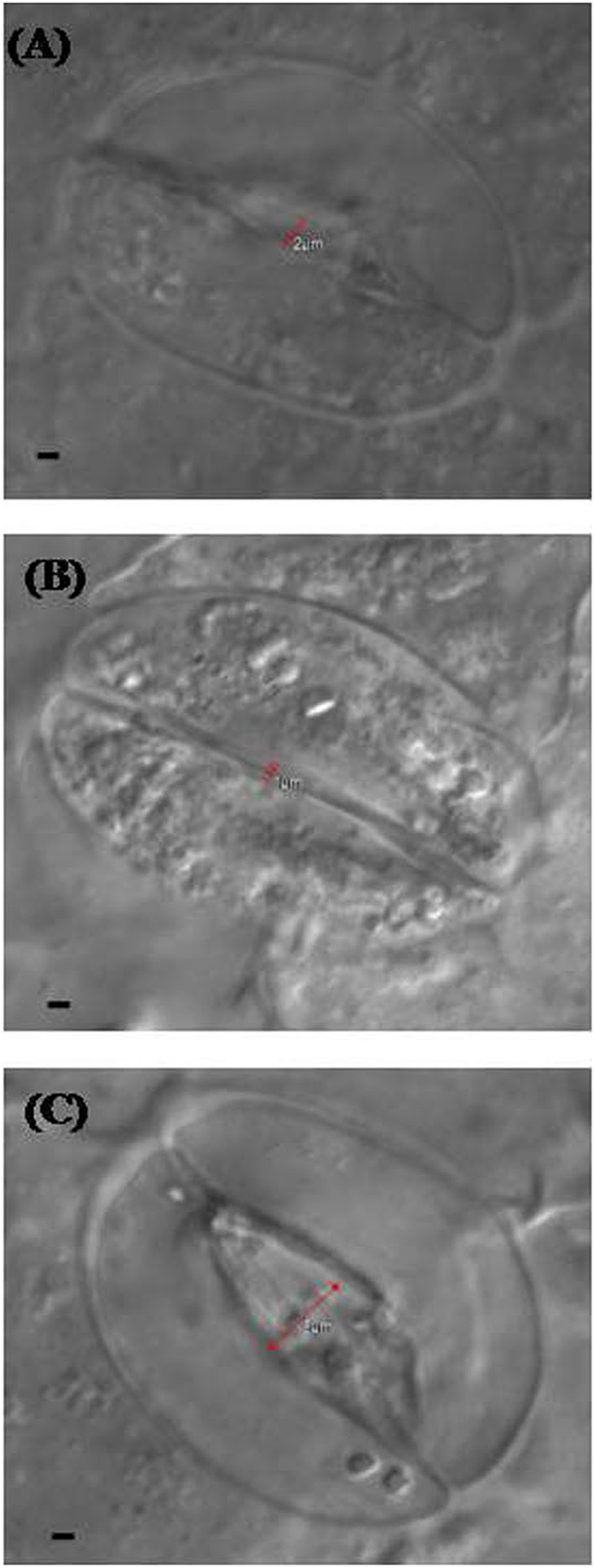
**Leaf stomatal response of mustard (***Brassica juncea*** L.) under the effect of various treatments**. The stomatal opening and closing response was studied using confocal microscopy in control **(A)**, 100 mM NaCl **(B)**, and S (200 mg S kg^−1^ soil; S) and 100 μM nitric oxide (NO) with 100 mM NaCl treated plants **(C)** at 30 DAS. Bar represents 1 μm in the panels **(A–C)**.

Confocal and electron microscopy were used to study the changes in guard cells in response to NO and S. Stomatal opening was 2 μm in diameter in leaf sample arising from control plants (Figure 5A), while it was slightly less or equal to 1 μm in diameter in leaf sample arising from salt grown plants (Figure 5B). Maximal stomatal opening of 5 μm diameter was found in leaf sample of plants grown with salt and treated with NO and S (Figure 5C).

**Figure 6:**
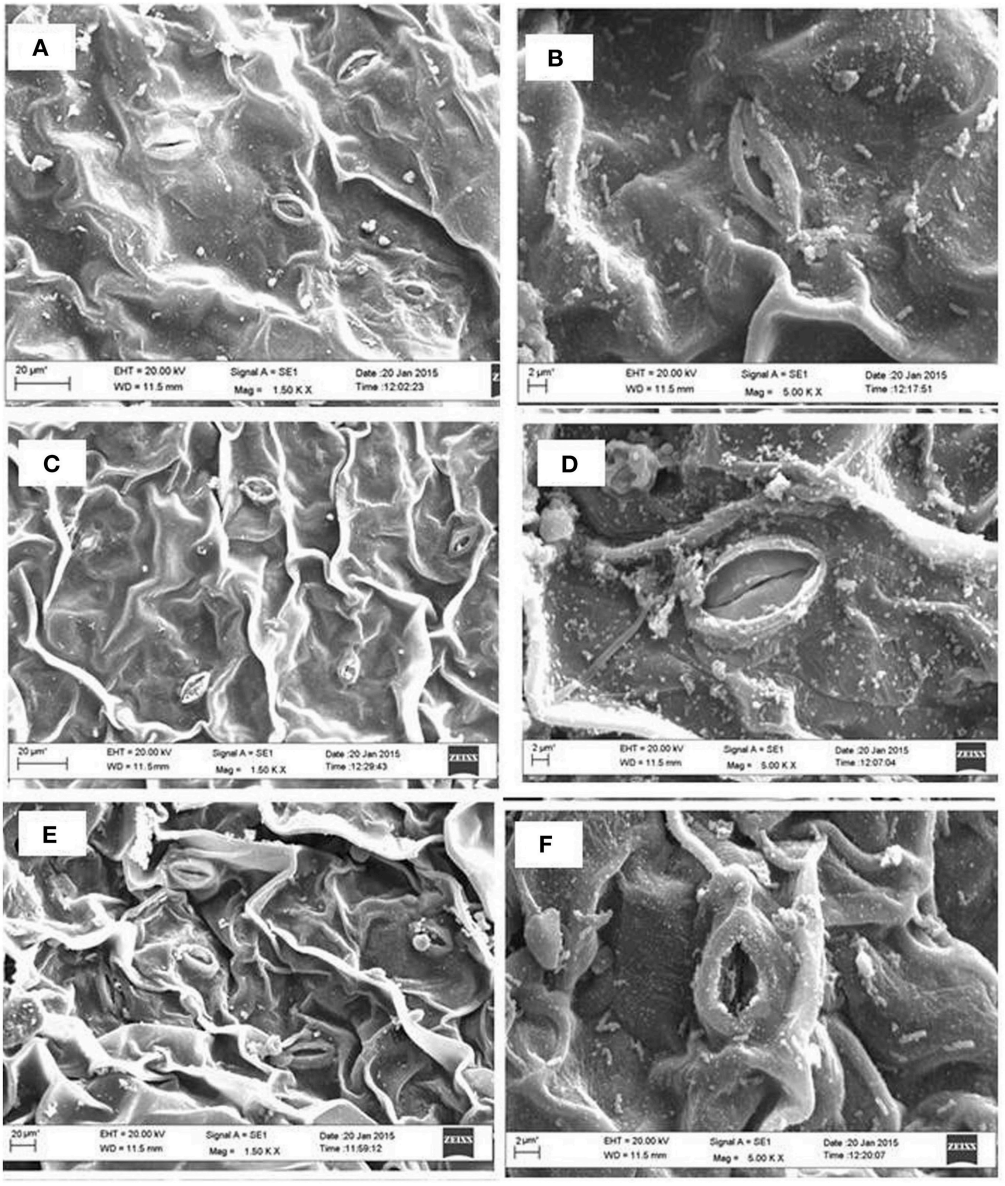
**Leaf stomatal behavior of mustard (***Brassica juncea*** L.) performed under control (A,B), 100 mM NaCl (C,D), and S (200 mg S kg^**−1**^ soil; S) and 100 μM nitric oxide (NO) with 100 mM NaCl (E,F)**. The effect of stomatal opening and closing was observed under the scanning electron microscopes at 1.50 K X **(A,C,E)** and 5.0 K X magnifications **(B,D,F)** in the leaf surface of mustard (*Brassica juncea* L.) grown under 100 mM NaCl treated plants at 30 DAS.

The analysis of SEM showed that partially closure of stomatal aperture in salt grown plants was prevented by the application of NO together with S. Moreover, the frequency of occurrence of stomata was higher in plants treated with NO and S than control or salt treated leaves (Figure 6). Salt stress decreased stomatal frequency by 25.0% compared to control plants. The plants supplemented with NO and grown with S reversed the effects of salt stress and increased the stomatal frequency by 39.2% in comparison to control plants.

#### Ultrastructural studies

**Figure 7:**
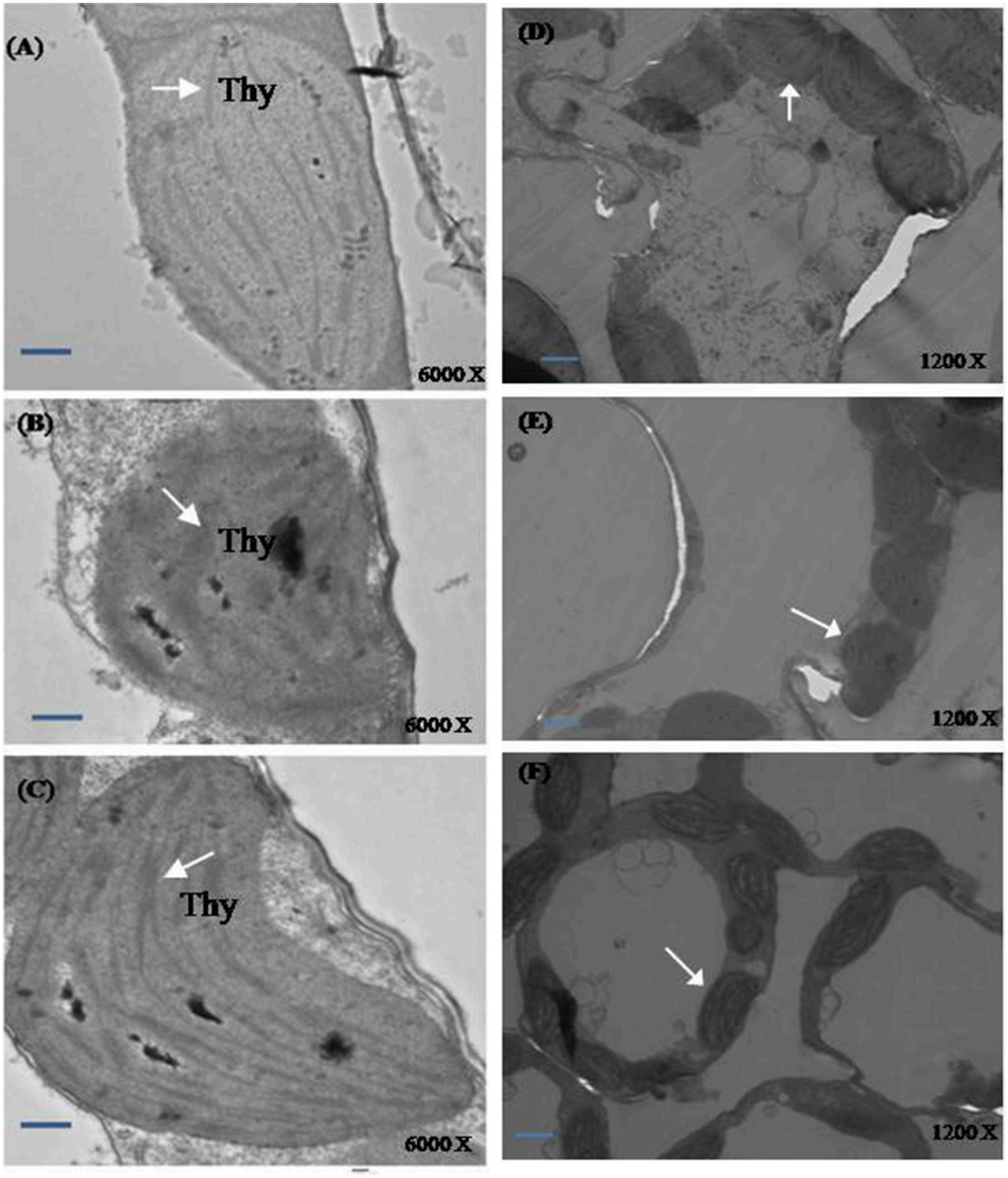
**Ultrastructure of chloroplasts from leaves of mustard (***Brassica juncea*** L.)**. Transmission electron microscopy micrographs on the representative chloroplasts from the leaves of mustard performed on the control **(A,D)**; 100 mM NaCl **(B,E)** and S (200 mg S kg^−1^ soil; S) and 100 μM nitric oxide (NO) with 100 mM NaCl treated plants **(C,F)** at 30 DAS. Ultrathin sections were prepared, stained with uranyl acetate and lead citrate, and examined by transmission electron microscopy operated at voltage of 120 kV and magnification of 6000 X and 1200 X. Bar represents 100 nm in the **(A–C)** and 500 nm in the **(D–F)**.(Thy; thylakoid membranes).

Plants grown under normal condition were characterized with well developed thylakoid membrane system (Figures 7A,D). However, greater modifications were seen in the salt treated plants. Plants grown with salt showed distorted chloroplast thylakoid system (Figures 7B,E). The more significant differences in chloroplast ultrastructure were observed in plants treated with NO and S under salt stress. The distortions of chloroplast structure by salt were seen lesser when plants received NO plus S. The chloroplast structure was well developed with regular shape and well-arranged thylakoid systems. A large number of thylakoid stacks were observed in the chloroplasts compared to control and salt treated plants (Figures 7C,F).

#### Effect of NO and S on growth characteristics

**Figure 8:**
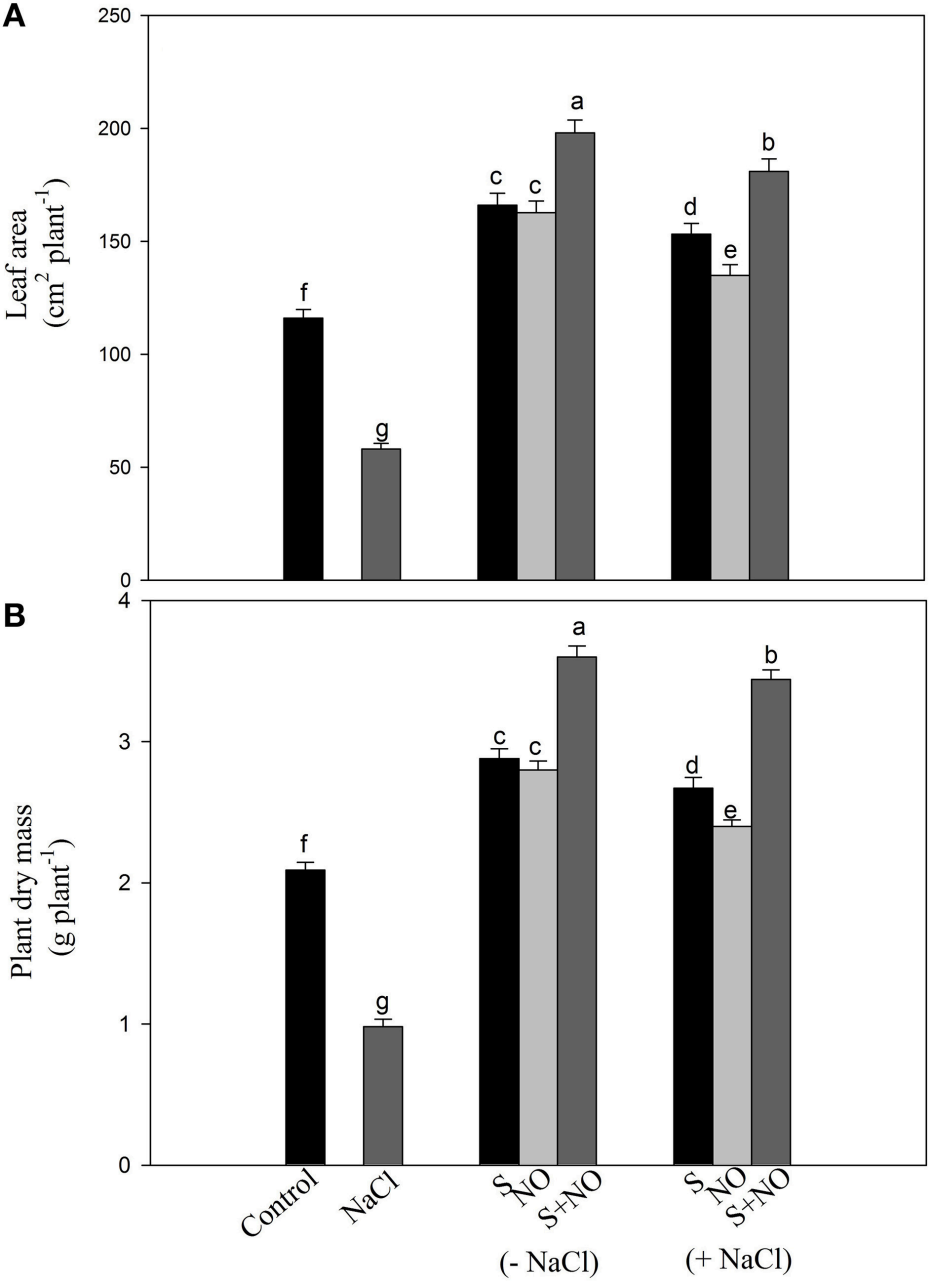
**Leaf area (A) and plant dry mass (B) of mustard (***Brassica juncea*** L.) treated with 100 μM nitric oxide (NO) and/or grown with S (200 mg S kg^**−1**^ soil; S) in presence or absence of 100 mM NaCl at 30 DAS**. Data are presented as treatments mean ± SE (*n* = 4). Data followed by same letter are not significantly different by LSD test at *P* < 0.05.

Salt stress decreased leaf area and plant dry mass by 49.9 and 53.1%, respectively with salt stress compared to control plants, but this inhibition was found reversed in plants receiving NO or S. The addition of NO or S increased leaf area equally by 43.1% and plant dry mass by 37.7%, respectively compared to control plants. Under salt stress, the effects of S was greater increasing leaf area and plant dry mass by 32 and 27.7%, while NO could increase leaf area and plant dry mass by 16.3 and 14.8%, respectively compared to control plants. The maximum increase in leaf area and plant dry mass was obtained with the combined treatment of NO and S compared to control plants under no stress (Figure 8).

## Discussion

### NO reduces ions accumulation and oxidative stress in plants receiving S

Plants treated with NO plus S accumulated Na^+^ and Cl^−^ in leaf and root to minimum under salt stress. The accumulation of ions in leaf was lesser than root showing that NO plus S treatment influenced the transport efficiency of ions. It has been shown that exogenous NO increased the activity of tonoplast H^+^ ATPase and Na^+^/H^+^ antiport facilitating Na^+^ compartmentation (Zhang Y. et al., 2006). The importance of NO in increasing the content of K^+^, Ca^2+^, and Mg^2+^ in salt-treated *Gossypium hirsutum* plants has been demonstrated (Dong et al., 2014a). It may be suggested that lowering of Na^+^ in cytosol by NO plus S treatment was through regulation of expression and activity of Na^+^ transporters and H^+^ pumps that generated the driving force for the transport. Application of NO plus S decreased H_2_O_2_ content and lipid peroxidation maximally and enhanced salt tolerance in plants by enhancing activity of the antioxidant enzymes. Recently Fatma et al. (2014), reported that S supplementation lowered ions accumulation and enhanced GSH production resulting in removal of H_2_O_2_ (Fatma et al., 2014). Moreover, NO has also the potential to modulate antioxidant defense system for scavenging ROS under salt stress (Kopyra and Gwozdz, 2003; Fatma and Khan, 2014). NO together with S facilitated the membrane functions through the induction of the antioxidant system and increased GSH production. Supplementation of NO or S to salt grown plants promoted activity of CAT, APX, and GR which were important for the efficient scavenging of H_2_O_2_ and TBARS. Bai et al. (2011) have reported that NO reinforces APX and CAT activities and reduces Cd-induced carbonylation in *Antiaris toxicaria* seeds. The involvement of NO has been demonstrated in protection of *Triticum aestivum* roots against aluminum toxicity (Sun et al., 2014). Further, NO up-regulated the activity and transcription of APX and GR, the two key enzymes in the AsA-GSH cycle in *Nicotiana tabacum* (Zhang et al., 2009a) and *Cucumis sativus* leaves (Cui et al., 2011) and conferred resistance to abiotic stress.

### NO reduces superoxide accumulation

It was found that application NO and S reduced the production of ROS (${\text{O}}_{2}^{\cdot -}$). The influence of NO and S on the inhibition of ROS assayed as superoxide accumulation has not been reported in the literature. The present study showed that application of NO and S on salt grown plants up-regulated the antioxidant system and prevented the accumulation of ROS. The role of NO has been recognized in detoxifying ROS either directly interacting with ${\text{O}}_{2}^{\cdot -}$ (Nakazawa et al., 1996) or enhancing function of the antioxidant system (Tewari et al., 2008). NO interacts with ROS such as ${\text{O}}_{2}^{\cdot -}$ and forms nitrating agent peroxynitrite, which serves as a signaling molecule in stress response and function in regulating protein activity (Baudouin, 2011). Bai et al. (2015) reported that NO-treated plants counteracted oxidative damage due to the decreased production rate of ${\text{O}}_{2}^{\cdot -}$ and reduced accumulation of H_2_O_2_.

### NO develops salt tolerance by enhancing S-assimilation

S-assimilation capacity of plants under salt stress was substantially increased with the combined application of NO and S resulting in increased activity of ATPS and content of Cys and GSH and redox state. The studies on the involvement of NO in enhancing S-assimilation in plants supplemented with S under salt stress have not been reported earlier. In this study, NO influenced S-assimilation by regulating the NO generation in plants receiving S. It has been reported that NO stimulates γ-glutamylcysteine synthetase and GSH synthetase gene expression in S-assimilation and up-regulates the GSH production in *Medicago truncatula* (Innocenti et al., 2007) and S. *lycopersicum* (Wang et al., 2015b). Under stress condition, excess GSH readily reacts with NO to form GSNO, which serves as NO reservoir in plants (Chaki et al., 2009; Wang et al., 2015a). The formed GSNO is catalyzed by GSNOR producing GSSG and NH_3_. The resulting GSSG is then reduced again to GSH by the NADPH-dependent reaction catalyzed by GR. Thus, a rapid recycling of GSH is related to the role of NO in maintaining the GSH pools as well as the ratio of GSH to GSSG. Hai-Hua et al. (2005) reported an increased GSH/GSSG ratio with exogenous application of NO in *T. aestivum* seedlings subjected to 150 mM NaCl stress. Exogenous NO increased GSH content in *S. lycopersicum* roots and leaves under copper (Cu) stress, adjusting the GSH/GSSG ratio (Wang et al., 2015b). Similarly, heat treatment resulted in decreased GSH/GSSG ratio which was reversed by the supplementation of NO (Hasanuzzaman et al., 2012). In *Oryza sativa* arsenic stress reduced GSH content and GSH/GSSG ratio and NO supplementation maintained the GSH/GSSG ratio (Singh et al., 2015). Additionally, the ability of exogenous NO to decrease lipid peroxidation has also been considered as one of the factors that maintains higher ATPS activity in *C. sativus* roots (Shi et al., 2007). Zhang et al. (2009b) showed that exogenous NO alleviated Cu toxicity by enhancing an antioxidative system and increasing ATPase activity in *S. lycopersicum*.

### The influence of NO and S on NO generation

The maximal NO generation was found in plants grown with salt stress. Gould et al. (2003) have also shown that NO generation increased in *N. tabacum* plants in response to salinity stress. The increased NO accumulation with exogenous NO and responses of plants to abiotic and biotic stresses suggested NO as an important signal molecule (Delledonne et al., 1998; Zhou et al., 2005). The application of NO and S increased the synthesis of NO and stimulated the activity of antioxidant enzymes for the protection from salt induced-oxidative stress. Neill et al. (2008) suggested that various stresses such as drought and salinity induced NO generation which activated cellular processes for protection against oxidative stress.

### NO and S influence ABA content, stomatal response, and chloroplast ultrastructure

Confocal and SEM analyses of plant samples revealed the potential role of NO and S in regulating the stomatal responses. Treatment of plants with salt induced stomatal closure and increased ABA accumulation, while application of NO and S together induced stomatal opening. These findings were also supported by the SEM images and also by much lesser increase in ABA in NO plus S treatment than salt treated plants. The stomatal frequency was also found higher with NO and S than both control and salt treated plants. In contrast to the present report that NO induced stomatal opening, Garcıìa-Mata and Lamattina (2001) found stomata closed in *Vicia faba* under drought stress. The NO induced stomatal opening in the present study might depend on osmotic relationship in plants cells. The influx/efflux of Ca^2+^ ions controlled stomatal movement (McAinsh et al., 2000) and ABA concentration in guard cells (Wang, 2014). Sakihama et al. (2003) reported that NO was involved in the signal transduction mechanisms for opening of stomata in *V. faba*. ABA induced increase in activity of antioxidant enzymes has been found linked with NO generation showing the relationship between ABA and NO in oxidative stress tolerance in *Stylosanthes guianensis* and *Arabidopsis thaliana* (Zhou et al., 2005; Neill et al., 2008). The present study also confirms the relationship between ABA and NO under salt stress. However, NO induced ABA content was found dependent on S that controlled stomatal movement. The increased GSH content with NO was further improved with S that was involved in cellular redox homeostasis and stomatal movement. The relation between ABA and S-assimilation has been shown earlier (Jiang and Zhang, 2001), where the authors showed ABA mediated accumulation of GSH. Besides, NO itself increased the antioxidant activity to protect plant from the oxidative stress induced by salt stress (Li et al., 2008). It is likely that potential interrelation between ABA, NO, and S-assimilation controlled the stomatal movement.

A coordinated influence of NO and S on the ultrastructural changes of chloroplasts under salt stress has not earlier been described. The increase in size of thylakoid with misshaped structure was prevented by the application of NO and S under salt stress. Chloroplast structure showed a well-developed and regular shape with well-arranged thylakoid systems. The plants exhibited more chloroplasts per cell or more thylakoid membranes per chloroplast than the control or salt treated plants with NO plus S due to the presence of higher chlorophyll content and lower level of lipid peroxidation.

### NO and S improve photosynthesis under salt stress

**Figure 9:**
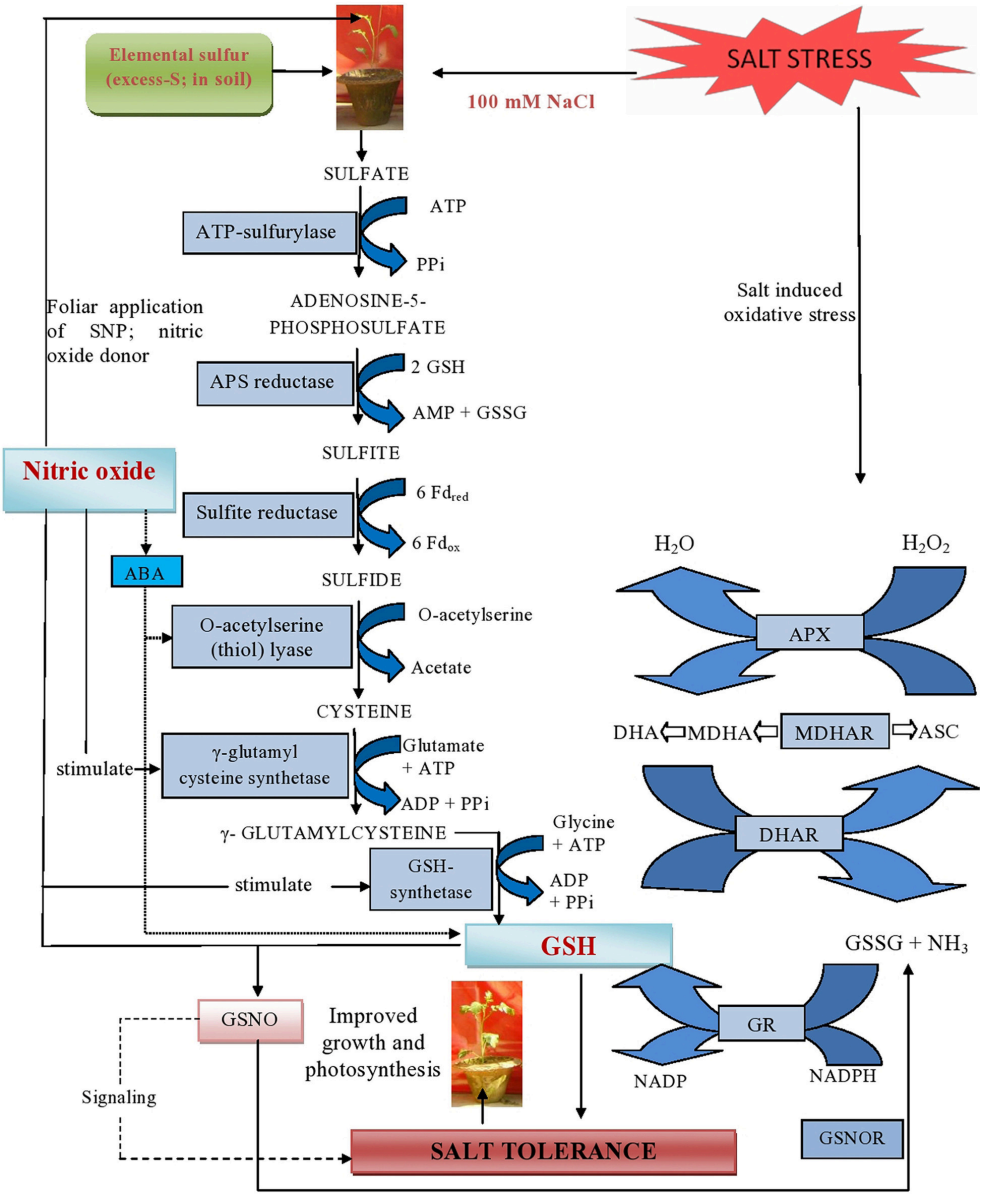
**Schematic representation of major mechanisms underlying excess-S and NO-mediated alleviation of salt stress in mustard (***Brassica juncea*** L.)**. The figure shows that NO generated by the supplementation of SNP (NO donor) can react with GSH and yield S-nitrosoglutathione (GSNO). This metabolite can be converted by the enzyme GSNO reductase (GSNOR) into GSSG and NH_3_. Additionally, supplementation of excess-S and NO reduced NaCl-induced oxidative stress and influenced photosynthesis by increasing GR-mediated conversion of GSSG into GSH and regulating NO generation in plants. Arrow (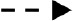) indicates signaling between NO and GSH for salt tolerance. Arrow (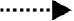) indicates interaction between NO and ABA. NO induces ABA accumulation which in turn up regulates S-assimilation and controls GSH formation.

Plants receiving exogenous NO and grown with S showed improved photosynthetic performance in the absence of salt. However, maximal increase in photosynthesis was noted with the combined treatment of NO plus S under both no stress and salt stress conditions (Table 1). Under salt stress, NO influenced photosynthesis by regulating NO generation in plants receiving S. The individual or combined application of NO and S favored S-assimilation, GSH synthesis, optimal NO generation and redox state. Moreover, there was also influence on the stomatal movement. The NO increased GSH production provided an important regulatory loop for NO bioactivity leading to better reduced cell environment under salt stress. Recently, it has been shown that application of NO increased photosynthesis through increase in stomatal conductance and Rubisco activity (Fatma and Khan, 2014). However, the increase in photosynthesis by exogenous NO and S under salt stress and the activity and SDS-PAGE profile of Rubisco and chlorophyll fluorescence are reported for the first time. Supplementation of NO and S was beneficial in increasing photosynthetic performance more than their individual effects because it maximally reduced the oxidative stress by increasing S-assimilation and antioxidants, and NO generation in suitable range. The involvement of NO in protection of chlorophyll against cadmium in *Helianthus annuus* (Laspina et al., 2005), drought stress in *O. sativa* (Farooq et al., 2009) and Cu in *Lolium perenne* (Dong et al., 2014b) has been reported. The application of NO has also resulted in improved gas exchange parameters and chlorophyll fluorescence under salt stress at seedling stage in *Lycopersicon esculentum* (Wu et al., 2011) and *H. vulgare* (Zhang L. et al., 2006). The response of NO has been linked with the availability of mineral nutrients. Wang et al. (2013) reported that NO increased uptake of Fe and magnesium was responsible for improving chlorophyll synthesis, photosynthesis and transpiration. Kong et al. (2014) reported that foliar application of NO and salicylic acid improved photosynthesis and supported Fe uptake, translocation and activation in *Arachis hypogaea*. Moreover, exogenous NO promoted the uptake and translocation of potassium, zinc and Fe mineral elements and stomatal aperture under Cu stress in *L. perenne* (Dong et al., 2014b). Figure 9 explains how the application of NO reduced salt induced oxidative stress and promoted photosynthesis in plants receiving S.

The present study showed clear differences in Rubisco protein pattern in plants receiving NO and S in salt treated plants. Protein bands with higher intensity were obtained in plants treated with NO plus S under salt stress. Fatma et al. (2014) showed that S application resulted in assemblage of new proteins and increased intensity of protein band in *B. juncea*.

### NO and S improve growth under salt stress

The inhibition of leaf area and plant dry mass was alleviated by the application of NO or S. Foliar spray of NO on various crops such as *Z. mays, O. sativa, L. esculentum* and *B. juncea* resulted in increased plant growth under salt stress that was due to high activity of antioxidant enzymes (Zhang et al., 2004; Farooq et al., 2009; Wu et al., 2011; Fatma and Khan, 2014). The study of Kausar et al. (2013) showed that the exogenous NO application proved beneficial in enhancing plant dry weight, length of both shoot and root of salt-stressed plants.

## Conclusion and future prospects

It may be concluded that NO or S improves photosynthetic performance of plants both under normal and salt stress conditions. Their combined application maximally alleviated salt stress induced effects on photosynthesis and growth of plants. The positive effect of the combined treatment of NO and S was through their influence on stomatal responses, S-assimilation and the antioxidant system. Thus, the utilization of S under salt stress was found as an essential key factor in activating the antioxidant system and GSH production. NO acted as signaling molecule which controlled the utilization of S and GSH production under salt stress. Therefore, their coordinated effect was more than their individual effect in alleviating salt stress and promoting photosynthetic and growth performance. Future strategies should be focussed to unravel the role of NO in regulating the various enzymatic steps of S-assimilation pathway and production of reduced S metabolites using molecular tools. It is also essential to examine the interaction between NO and other phytohormones in signaling stomatal response and regulation of photosynthesis. It is known that S-assimilation is linked to ethylene production through Cys synthesis and NO promotes S-assimilation. It is therefore, likely that NO and S regulated interaction between ABA and ethylene in guard cells that may increase the stomatal and photosynthetic response under salt stress although this remains to be tested.

## Author contributions

MF designed and conducted the experiment, TP, AM helped in data analysis and presentation, while NK overall supervised the work and corrected the manuscript.

### Conflict of interest statement

The authors declare that the research was conducted in the absence of any commercial or financial relationships that could be construed as a potential conflict of interest.
